# Sleep problems are related to commuting accidents rather than to workplace accidents

**DOI:** 10.1186/s12889-021-10737-5

**Published:** 2021-04-06

**Authors:** Héctor Vargas-Garrido, Emilio Moyano-Díaz, Katherinne Andrades

**Affiliations:** Faculty of Psychology, Psychology Department, University of Talca, Talca, Chile

**Keywords:** Commuting accidents, Sleep quantity, Sleep quality, Workplace accidents

## Abstract

**Background:**

This study aimed to verify the relationships between sleep problems and both commuting and workplace accidents in workers of both sexes.

**Methods:**

The study was carried out with a sample of workers (*n* = 2993; 50.2% female) from the Chilean Quality of Life Survey (ENCAVI) 2015–2016, while the rates of both workplace and commuting accidents were extracted from the statistics of the Superintendence of Social Security (SUSESO 2015; 180,036 and 52,629 lost-time accidents, respectively).

**Results:**

Chilean workers sleep less than the rest of the people in the country (*M*_W_ = 7.14 vs. *M*_O_ = 7.33; *t* (6789) = − 5.19; *p* < .001), while the Chilean people as a whole sleep less compared to those of other countries (7.24 h per day). Likewise, it was found that sleep problems are more strongly related to commuting than to workplace accidents. In this vein, sleep quantity can explain 24% of the variance in commuting accidents’ rates (Stepwise Method; *R*^2^ = .30, *F* (1.14) = 5.49, *p* < .05; *β* = −.55, *p* < .05), by using aggregated data with all types of commuting roles (driver of a vehicle, a passenger of public or private transport, or as a pedestrian).

**Conclusions:**

Our findings show that sleep quantity has a more robust relationship with commuting than workplace accidents, a neglected issue so far. Future prevention programs should emphasize sleep hygiene and focus on commuting to and from work.

**Supplementary Information:**

The online version contains supplementary material available at 10.1186/s12889-021-10737-5.

## Background

Two types of occupational accidents workers can be involved in: at the workplace and while commuting. A workplace accident is any physical injury suffered by an individual caused or as a result of working, while a commuting accident takes place on the way to and from the place of work [1]. These two types of accidents have noticeable differences. While workplace accidents decrease yearly, commuting accidents increase [2–4]. By sex, more men are involved in workplace accidents while women in commuting accidents [2, 5]. Global data indicate that commuting accidents have more severe consequences of death and permanent disability than workplace accidents and imply higher economic costs [6]. Likewise, commuting accidents are legally treated in diverse ways worldwide [7]. They are covered by insurance in Chile as in Greece, Germany, and Brazil, but not in Denmark, Poland, and the USA [6]. Also, European statistics on occupational accidents [8] expressly exclude commuting accidents, unlike Chile, where they are part of the social security statistics. These different regulatory treatments of commuting accidents by countries contrast from the more homogeneous treatment of workplace accidents. Thus, workplace and commuting accidents exhibit different behaviors and characteristics and are also treated differently by countries’ legislation and statistical methodologies.

Despite their worse effects and persistence over time, commuting accidents have not received deep attention [4, 9, 10]. For example, most research regarding sleep problems has been centered on workplace accidents (including professional drivers’ accidents), and less when workers drive to and from their workplace (neglecting other commuting roles such as pedestrian or passenger) [11–14].

Research usually considers two dimensions of sleep: quantity and quality. Sleep quantity is defined as the amount of time someone spends sleeping [15]. Meanwhile, sleep quality usually refers to variables such as insomnia (not sleeping), poor sleep quality (not restful), and the need to use sleep medications, among others [11, 16]. It is worth noting that 7 hours is an appropriate amount of nighttime sleep for adults to maintain adequate levels of health and cognitive functioning [15]. However, few sleep hours and long working hours are considered normal in modern societies [17, 18]. For example, Komada et al. [19] reported that in the 1960s, 90% of Japanese people went to sleep at 11 p.m. and for periods of at least 8 h, while in 2010 more than half of the people were still awake by that time and slept an average of 7 h and 14 min. These antecedents could be associated with the observed chronic lack of sleep in developed countries, a situation that leads to excessive daytime sleepiness [12, 20, 21]. Sleeping less than 7 hours or having sleep quality disturbances are linked to several risks, such as heart disease, obesity, diabetes, hypertension, a weaker immune defense system, a lower cognitive performance, adverse effects on mental health, depression, burnout, and in general, all kinds of causes of death [11, 17, 22]. However, people with sleep problems habituate themselves and underestimate their sleepiness levels and poor sleep quality [23]. Stronger relationships between sleep quantity and quality have been observed when both variables are measured using subjective or self-report methods, and less intense relationships when at least one of these variables is objectively measured [13].

Concerning sleep quality, Uehli et al. [11], in a systematic review and meta-analysis of 27 studies, showed that workers with sleep problems are at higher risk of injury by accidents compared to those without sleep problems. Nearly 13% of workplace injuries can be attributed to sleep problems. The use of medicine against sleep disturbances and breathing problems while sleeping are the most adverse factors; meanwhile, daytime sleepiness has the lowest effect among studied sleep problems. Likewise, fatigue and drowsiness have been associated with work-related traffic injuries [24].

Regarding sleep quantity, sleeping less than 6 hours is a risk factor for traffic accidents in all drivers [19] and professional truck drivers [12]. Additionally, Carter et al. [25] found that traffic accidents, either recreational or commuting to work, and workplace accidents, were related to lack of sleep using self-report accidents among Swedish men. Similarly, the lack of sleep has been linked to impaired driving in simulated commuting trips [14]. Likewise, it has also been observed that sleeping less than the suggested number of hours is a risk factor linked to workplace accidents in the construction industry [26] and among civil servants [27].

In sum, deficits in sleep quantity and quality—with their adverse effects on people’s normal functioning and health—are linked to either workplace and commuting accidents [21, 28]. However, this latter association is specifically when workers drive to or from work [25]. Nevertheless, comparing the strength of the relationships between sleep problems and both types of accidents remains an issue. In this study, the central question is whether sleep problems are related to workplace accidents as the same as commuting accidents. We consider that commuting and workplace accidents have different characteristics, so we hypothesized differences in the strength of the relationships between sleep problems and both types of accidents. By analyzing two secondary sources of information, we test this supposition. We linked sleep quantity and quality of workers [29] with both workplace and commuting accidents [30]. It has to be noted that this work considers aggregated data with all types of commuting accidents, namely when the worker is the driver of a vehicle, a passenger, or a pedestrian going to or from their workplaces.

## Material and methods

### Source of information

In Chile, there is not a single database that simultaneously contains all the information we need. Then, as a proxy for exploration, we use two independent databases: ENCAVI 2015–2016 for both sleep quantities and sleep quality [29], and the statistics of the Superintendence of Social Security (SUSESO) for workplace accidents and commuting accidents rates [30]. ENCAVI 2015–2016 and SUSESO’s statistics are used in research as secondary data sources [31, 32].

ENCAVI 2015–2016 includes 7041 respondents nationwide, representing people over the age of 15 living in private homes located in urban and rural areas of the 15 regions of Chile. Interviewers applied this paper-and-pencil survey in a face-to-face procedure at the chosen household. Through a probabilistic, geographically stratified, and multi-stage design (four stages: district, city block, housing, and person), houses were chosen. To homogenize the type of participants in both databases, only employees were considered from the ENCAVI 2015–2016 in this study (*n* = 2993; 50.2% women; average age = 43.3 years), which excluded pensioners, students, homemakers, among others. Sleep quantity and sleep quality were extracted from the topic ‘Sleep Hygiene’ of Module III, ‘Well-being and Health Perceptions,’ of ENCAVI 2015–2016. The question about the amount of sleep is this: ‘How many hours do you estimate you have slept, on average, each night during the last month?’ The six questions related to sleep quality are the following: i) Sleep quality in the last month (1 = Quite good, 2 = Good, 3 = Bad, 4 = Quite bad); ii) Use of medicines for sleeping in the last month (1 = None, 2 = Less than once per week, 3 = Once or twice per week, 4 = Three or more per week); iii) Incidents of sleepiness in the last month (1 = None, 2 = Less than once per week, 3 = Once or twice per week, 4 = Three or more per week); iv) Problems such as falling asleep during daytime, waking up frequently at night, or waking up too early in the morning during the last 30 days (1 = None, 2 = Rarely, 3 = Not much, 4 = A lot, 5 = Too much); v) Difficulty in feeling rested and refreshed during the day (1 = None, 2 = Rarely, 3 = Not much, 4 = A lot, 5 = Too much); and vi) Sleep quality in the last year (1 = Quite bad, 2 = Bad, 3 = Neutral, 4 = Good, 5 = Quite good) [29]. The ENCAVI database is for public use and was obtained from the Ministry of Public Health of Chile. The implementing agency of the Encavi Survey (Tender No. 757–7-LP15) was the Department of Social Studies (DESUC, Institute of Sociology) of the Pontifical Catholic University of Chile (PUC). DESUC team applied informed consent to all participants.

SUSESO’s statistics summarize 180,036 workplace accidents and 52,629 commuting accidents that generated lost time, covering 4,832,489 all insured workers nationwide split by the 15 regions of Chile, with national rates of 3.73 and 1.09%, respectively. SUSESO’s commuting accidents include all possible workers’ roles: a driver of a vehicle (cars, motorcycles, bikes, scooters, among others), a passenger (public and private transport), or a pedestrian [30].

Both ENCAVI 2015–2016 and SUSESO’s statistics contain national records corresponding to year 2015 in their respective topics.

### Plan of analysis

Both databases come from the same population, but individuals’ identification was not possible. So, we merged both databases taking the 15 regions of Chile as units of analysis, also differentiating by sex within each area. Correlations between average scores of sleep quantity and sleep quality with the rates of workplace and commuting accidents among Chilean workers, by region, were calculated using the SPSS 21. Regression analyses were performed on the workplace and commuting accident rates with sleep variables as predictors.

## Results

Analysis of correlations between average scores of sleep quantity and sleep quality with the rates of workplace and commuting accidents among Chilean workers, by region, showed significant negative correlations between commuting accidents with ‘sleep hours’ in the total sample, *r* (15) = −.55; *p* < .05; and in the case of males, *r* (15) = −.52; *p* < .05 (see Table 1). However, ‘sleep hours’ did not correlate with workplace accidents for the sample as a whole or when differentiating by sex.

**Table 1:** Pearson correlations among sleep variables with commuting and workplace accidents’ rates of chilean workers

	Workplace Accidents				Commuting Accidents				
	Males		Females	Total	Males		Females	Total	
Quantity									
Sleep Hours	−.239		.067	−.224	−.520	*	−.278	−.545	*
Quality									
S. Quality (LM)	.460	#	−.416	.287	.397		−.200	.186	
Use of Medic.	.029		.190	−.075	−.435		−.308	−.369	
Sleepiness	.297		−.327	.340	.446	#	−.149	.227	
Sleep problems	.403		−.316	.290	.362		−.060	.275	
Dif. Feel. Rested	.437		−.401	.247	.370		−.188	.173	
S. Quality (LY)	−.323		.293	−.249	−.312		.190	−.156	

*p < .05; #*p* < .10

Regarding sleep quality variables, marginally significant correlations were found in the case of males between ‘sleep quality (last month)’ with workplace accidents, *r* (15) = .46; *p* = .08; and ‘sleepiness’ with commuting accidents, *r* (15) = .45; *p* = .09.

To check whether the sleep quantity variable and the six indicators of sleep quality could explain the accident rates, linear regression models were performed on the workplace and commuting accident rates, with the seven sleep variables as predictors. Two regressions fit adequately into a model: ‘sleep hours’ as a predictor of commuting accident rates for the whole sample (see Table 2) and ‘sleep hours’ as a predictor of commuting accident rates for males.

**Table 2:** Linear regression model on commuting accidents and sleep variables as predictors for Workers of 15 Chilean Regions

	B	SE B	β	t
Constant	3.20	1.00		3.19**
Sleep hours	−.32	.14	−0.55	−2.34*

Adjusted *R*^*2*^ = .24 (Stepwise Method; *R*^*2*^ = .30, *F* (1,14) = 5.49, *p* < .05)

*p < .05;** *p* < .01

Table 2 shows that sleep hours can explain 24% of the variance in commuting accident rates in the total sample. When only men were considered, the variance explained reached 21% (Stepwise Method; *R*^2^ = .27, *F* (1.14) = 4.79, *p* < .05; *β* = −.52, *p* < .05). Therefore, these results allow us to conclude that there were significant relationships between sleep hours and commuting accidents, but not with accidents at the workplace.

Additionally, for comparative purposes, regressions models using the enter method, show that sleep quality problems in males have similar strength to explain the variance of both workplace, *R*^2^_Adjusted_ = .15, *F* (1.14) = 3.50, *p* = .08; *β* = .46, *p* = .08; and commuting accidents, *R*^2^_Adjusted_ = .14, *F* (1.14) = 3.24, *p* = .09; *β* = .45, *p* = .09 (marginal effects).

## Discussion

The main finding is that hours of sleep are significantly related to commuting accident rates by region of the country, and it can explain 24% of their variance. That is, as workers in a region sleep lower, higher rates of commuting accidents in the region are found (see Annex 1). This raises an essential question about causation: are detriments in sleep quantity causing commuting accidents, or are worse sleep hours a by-product of having had commuting accidents? Although our research does not clarify the direction of the relationship, robust previous evidence shows that sleep problems are a predisposing factor for accidents [11, 19, 24, 25, 27].

Our findings are in line with previous research indicating that sleep quantity’s detriments have negative implications for the accidents that workers have as they drive to work [14, 25]. However, this research expands the knowledge because it considers information aggregated with all types of commuters roles, not only when the worker is the driver but also when he/she is a passenger or a pedestrian; likewise, it takes into account accidents with lost time. We observed this main effect within the total sample and with male workers is also in line with previous literature observing that sleep problems and casualties have stronger relationships for males than for females [24, 33].

Nevertheless, unlike previous evidence [25–27], sleep quantity of Chilean workers seem to be unrelated to workplace accidents. It is worth noting that previous works have used different research procedures or show small effects. Indeed, Carter et al. [25] asked for self-reported workplace accidents among their male Swedish participants—regardless of severity, while we used statistics of accidents with lost time for both males and females. Powell and Coping [26] stated that “less sleep may be a factor” for workplace accidents in the construction workers, “but the statistical significance is weak (*p* = 0.22)”. Likewise, Salminen et al. [27] found that civil servants with short sleep (< 7 h) are involved in occupational injuries 1.25 times more than those who sleep between 7—8 h. In this study, we have used information about workers nationwide from all economic sectors. Therefore, these antecedents seem to show— more generally, a less stable relationship between sleep quantity and workplace accidents, and this relationship becomes more potent in some specific contexts, such as type of industry or occupation.

Regarding sleep quality, although we have found marginally significant effects only in the case of males, they are in line with previous findings, which allows inferring they are not at random. Firstly, ‘sleep quality’ problems related to workplace accidents were verified by Uehli et al. [11]. Secondly, although there is no specific previous evidence on ‘sleepiness’ related to commuting accidents, closest studies have linked sleepiness with work-related road accidents [12, 24]. Nevertheless, sleep quality problems in males seem to have similar strength with both workplace and commuting accidents.

Thus, our results for the whole sample combined with previous findings suggest that sleep problems, specifically sleep quantity have stronger relationships with commuting than workplace accidents, a neglected issue. More attention is needed on sleep problems, and workers’ health surveillance [34, 35], and our findings shed light on future prevention programs.

Which factors could then be related to the fact that detriment in sleep quantity affects workers’ accidents more strongly while they commute to work than at the workplace? Without a definitive answer, several factors should be considered.

First, sleep inertia is a transitional stage of low activation that occurs after waking up and temporarily generates a decrease in performance. This phenomenon gets worse when there is a lack of sleep and can have effects up to 4 hours after awakening [36]. Sleep inertia would be consistent with the fact that commuting accidents occur largely in the morning rather than in the afternoon [5, 10]. Simulated driving studies have found that sleep inertia impairs the performance of sleep-deprived drivers [37], mainly when they drive in the morning rather than in the afternoon [14]. In this line, Chileans have antecedents of sleep detriment that could make sleep inertia worse. For example, Chilean workers sleep significantly less than other people in the country, *M*_W_ = 7.14 vs. *M*_O_ = 7.33; *t* (6789) = − 5.19; *p* < .001 (ENCAVI 2015–2016). Likewise, the Chilean population, in general, seems to sleep less than those of other countries. In a multi-country comparison, Walch et al. [38] found that the longest sleep patterns are among people in the Netherlands and New Zealand (slightly more than 8 h), while the shortest are for people in Japan and Singapore (bordering on 7.5 h), still exceeding the average for Chileans (7.24 h) [29].

Second, some workplace conditions can interfere with sleep. Sleep disturbances can, in turn, endanger the safety of workers [39]. For example, both work-family conflict and inadequate supervision affect workers’ safety when they drive to work [9], as do the pressures associated with work deadlines [40] and to get to the workplace on time, especially when rewards or punishments are involved. Likewise, low social status or income, shift work-, and work-related stress are often associated with sleep problems [21, 41].

Third, a situational factor comprises those of the traffic context. These include the poor condition of streets and sidewalks, street dogs, crime and vandalism, reckless behaviors by other drivers, cyclists, and pedestrians, among others [42].

All in all, we can conclude that workers with lack of sleep, while commuting to work, are subject to a series of adverse factors (sleep inertia, workplace conditions that interfere with sleep, and environmental situations on the streets) that seem to interact with the impaired normal functioning that sleep detriment implies [12, 28, 34, 39, 41, 43], producing a more unfavorable scenario for accidents on the way to or from work rather than at the workplace. In this vein, reduced alertness is one of the main consequences of sleep restriction [43, 44], and commuting is a substantial attention-demanding task which, in turn, becomes most sensitive to reduced alertness. At the workplace, employees supervise processes taken over by machines and computers, or perform tasks themselves in a steadier and more predictable context than those on the streets. Therefore, lapses of attention may be relatively marginal causes of workplace accidents compared to the more critical role played in commuting contexts.

Likewise Uehli et al. [11] pointed out that workers with sleep problems are aware of their limitations; therefore, they generate coping strategies at workplaces where they find greater control and predictability. In this regard, workers’ coping strategies can be expected to be limited or restricted within commuting contexts, where they have less control over the intervening factors and are more affected by reduced alertness.

Among the limitations of the present work, the main one is using data from two different databases. Although the information comes from the same workers grouped by the country’s regions, any specific worker identification was impossible. In this vein, there might be individual differences in sleep patterns that are not considered and other not controlled factors, such as climatic conditions, traffic environments, etc. However, our findings are consistent with previous literature and shed light on more accurate and sophisticated future research procedures.

Likewise, the reliability of the measurement of sleep hours by self-report could be questioned. However, evidence found through meta-analysis shows that a subjective or self-report measure strongly correlates with objective measures of the amount of sleep [13]. Likewise, those correlations are more robust than correlations between objective and subjective measures of sleep quality. Nevertheless, this limitation offers a chance to continue working in this line of research by using different measurements of sleep quantity and sleep quality.

Also, we have used aggregated information with all workers’ commuting roles (driver, passenger, or pedestrian). There might be differences in the strength of relationships if those roles were studied separated. Nevertheless, commuting accidents have received few attention [4, 9, 10], and solely considering workers as drivers. So, this is a first step to go further exploring those other neglected roles (pedestrians or passengers).

To conclude, global data indicate that commuting accidents have more severe consequences and imply higher economic costs than workplace accidents [6]. However, most research regarding sleep problems has been centered on workplace accidents [11–14]. In this work, by comparing lost time accidents and using aggregated data with all types of commuting roles, we have found that sleep problems, specifically sleep quantity have stronger relationships with commuting than workplace accidents, a neglected issue so far. Future prevention programs should emphasize sleep hygiene and focus on commuting to and from work.

## Supplementary Information

**Additional file 1: Annex 1**. Workers’ Sleep Variables of ENCAVI 2015-2016 and Rates of Workplace and Commuting Accidents of SUSESO 2015, per sex and region of Chile

## Data Availability

The SUSESO database analyzed during the current study are available in the SUSESO statistics repository, https://www.suseso.cl/608/w3-article-19276.html. The ENCAVI 2015–2016 is asked under request to the Ministry of Public Health.
