# Supplementary material for: Sleep problems are related to commuting accidents rather than to workplace accidents
Source: BMC Public Health. 2021 Apr 6;21:652. doi: 10.1186/s12889-021-10737-5 (PMC8022368; doi:10.1186/s12889-021-10737-5)
Supplement: Supplementary file 1 — Additional file 1: Annex 1. Workers’ Sleep Variables of ENCAVI 2015-2016 and Rates of Workplace and Commuting Accidents of SUSESO 2015, per sex and region of Chile [file 12889_2021_10737_MOESM1_ESM.docx]

Annex 2

Workers’ Sleep Variables of ENCAVI 2015-2016 and Rates of Workplace and Commuting Accidents of SUSESO 2015, per sex and region of Chile

|  | Sleep Variables of  ENCAVI 2015-2016 | | | | | | | | | | | | | | | | | | | | | |  | Occupational Accidents Rates 2015 | | | | | | |
| --- | --- | --- | --- | --- | --- | --- | --- | --- | --- | --- | --- | --- | --- | --- | --- | --- | --- | --- | --- | --- | --- | --- | --- | --- | --- | --- | --- | --- | --- | --- |
|  |  |  |  |  |  |  |  |  |  |  |  |  |  |  |  |  |  |  |  |  |  |  |  |  |  |  |  |  |  |  |
|  | Quantity | | |  | Quality | | | | | | | | | | | | | | | | | |  | Workplace | | |  | Commuting | | |
|  |  |  |  |  |  |  |  |  |  |  |  |  |  |  |  |  |  |  |  |  |  |  |  |  |  |  |  |  |  |  |
|  | Sleep Hours | | |  | S. Quality (LM) | | | Use of Medic. | | | Sleepiness | | | Sleep problems | | | Dif. Feel. Rested | | | S. Quality (LY) | | |  |  |  |  |  |  |  |  |
|  | H | M | T |  | H | M | T | H | M | T | H | M | T | H | M | T | H | M | T | H | M | T |  | H | M | T |  | H | M | T |
|  |  |  |  |  |  |  |  |  |  |  |  |  |  |  |  |  |  |  |  |  |  |  |  |  |  |  |  |  |  |  |
| ARIC | 7.13 | 7.02 | 7.08 |  | 2.00 | 2.00 | 2.00 | 1.35 | 1.61 | 1.47 | 1.43 | 1.44 | 1.43 | 1.63 | 1.44 | 1.54 | 1.65 | 1.67 | 1.66 | 3.77 | 3.63 | 3.71 |  | 5.56 | 4.15 | 4.94 |  | 1.01 | 1.27 | 1.12 |
| TARA | 7.40 | 7.16 | 7.29 |  | 1.87 | 2.07 | 1.96 | 1.24 | 1.33 | 1.28 | 1.27 | 1.43 | 1.34 | 1.32 | 1.66 | 1.47 | 1.40 | 1.91 | 1.63 | 3.93 | 3.60 | 3.78 |  | 3.83 | 3.61 | 3.76 |  | 0.47 | 1.44 | 0.80 |
| ANTO | 7.79 | 7.99 | 7.90 |  | 1.27 | 1.25 | 1.26 | 1.00 | 1.14 | 1.07 | 1.07 | 1.11 | 1.09 | 1.11 | 1.10 | 1.10 | 1.16 | 1.17 | 1.16 | 4.78 | 4.71 | 4.74 |  | 2.71 | 4.40 | 3.22 |  | 0.51 | 1.51 | 0.82 |
| ATAC | 7.68 | 7.16 | 7.48 |  | 1.80 | 2.00 | 1.88 | 1.74 | 2.42 | 2.00 | 1.27 | 1.29 | 1.28 | 1.27 | 1.63 | 1.41 | 1.48 | 1.72 | 1.57 | 3.87 | 3.63 | 3.78 |  | 2.61 | 4.00 | 3.06 |  | 0.44 | 0.97 | 0.61 |
| COQU | 6.98 | 6.92 | 6.95 |  | 1.95 | 1.90 | 1.93 | 1.25 | 1.43 | 1.34 | 1.51 | 1.77 | 1.63 | 1.57 | 1.81 | 1.69 | 1.58 | 2.16 | 1.88 | 3.74 | 3.70 | 3.72 |  | 3.37 | 2.95 | 3.22 |  | 0.49 | 1.04 | 0.69 |
| VALP | 6.74 | 6.78 | 6.76 |  | 2.05 | 2.15 | 2.10 | 1.12 | 1.36 | 1.24 | 1.59 | 1.85 | 1.72 | 1.65 | 1.98 | 1.81 | 1.73 | 2.04 | 1.88 | 3.71 | 3.37 | 3.54 |  | 5.34 | 4.33 | 4.97 |  | 0.88 | 1.79 | 1.22 |
| OHIG | 7.20 | 7.06 | 7.13 |  | 1.80 | 2.01 | 1.92 | 1.12 | 1.63 | 1.40 | 1.24 | 1.48 | 1.37 | 1.23 | 1.64 | 1.45 | 1.38 | 1.92 | 1.68 | 4.08 | 3.72 | 3.88 |  | 5.02 | 4.36 | 4.79 |  | 0.66 | 1.06 | 0.80 |
| MAUL | 7.06 | 7.09 | 7.08 |  | 1.99 | 2.15 | 2.08 | 1.07 | 1.48 | 1.30 | 1.46 | 1.73 | 1.61 | 1.46 | 1.63 | 1.55 | 1.43 | 1.86 | 1.67 | 3.75 | 3.44 | 3.58 |  | 4.84 | 3.52 | 4.36 |  | 0.62 | 1.07 | 0.79 |
| BIOB | 7.84 | 7.55 | 7.69 |  | 1.79 | 1.85 | 1.82 | 1.16 | 1.41 | 1.29 | 1.14 | 1.29 | 1.21 | 1.31 | 1.40 | 1.36 | 1.41 | 1.55 | 1.48 | 4.07 | 3.97 | 4.02 |  | 3.68 | 2.56 | 3.28 |  | 0.55 | 1.04 | 0.72 |
| ARAU | 6.85 | 6.90 | 6.88 |  | 2.10 | 2.24 | 2.17 | 1.31 | 1.32 | 1.32 | 1.36 | 1.38 | 1.37 | 1.73 | 1.68 | 1.70 | 1.71 | 2.00 | 1.87 | 3.63 | 3.69 | 3.66 |  | 4.81 | 2.86 | 4.14 |  | 0.66 | 1.14 | 0.82 |
| RIOS | 8.13 | 7.60 | 7.92 |  | 1.90 | 2.20 | 2.02 | 1.30 | 1.20 | 1.26 | 1.23 | 2.12 | 1.58 | 1.23 | 1.88 | 1.48 | 1.43 | 2.08 | 1.68 | 4.05 | 3.52 | 3.85 |  | 5.59 | 2.85 | 4.62 |  | 0.55 | 0.85 | 0.65 |
| LAGO | 7.03 | 7.08 | 7.05 |  | 1.83 | 1.88 | 1.86 | 1.54 | 1.55 | 1.55 | 1.30 | 1.51 | 1.40 | 1.47 | 1.53 | 1.50 | 1.57 | 1.57 | 1.57 | 4.00 | 3.87 | 3.94 |  | 5.25 | 3.36 | 4.60 |  | 0.44 | 1.06 | 0.65 |
| AISE | 7.65 | 7.78 | 7.72 |  | 1.81 | 1.79 | 1.80 | 1.80 | 1.80 | 1.80 | 1.35 | 1.67 | 1.53 | 1.67 | 1.84 | 1.77 | 1.73 | 2.03 | 1.90 | 4.00 | 3.75 | 3.86 |  | 5.10 | 3.66 | 4.50 |  | 0.29 | 1.06 | 0.61 |
| MAGA | 7.33 | 7.07 | 7.21 |  | 1.74 | 1.75 | 1.74 | 1.21 | 1.43 | 1.30 | 1.25 | 1.41 | 1.32 | 1.42 | 1.50 | 1.46 | 1.53 | 1.60 | 1.56 | 4.10 | 4.07 | 4.09 |  | 5.23 | 4.67 | 5.02 |  | 0.88 | 1.91 | 1.27 |
| SANT | 6.80 | 6.62 | 6.71 |  | 2.06 | 2.20 | 2.13 | 1.24 | 1.51 | 1.38 | 1.52 | 1.74 | 1.64 | 1.65 | 1.92 | 1.79 | 1.75 | 2.09 | 1.93 | 3.75 | 3.48 | 3.61 |  | 3.93 | 2.79 | 3.48 |  | 1.01 | 1.56 | 1.23 |
|  |  |  |  |  |  |  |  |  |  |  |  |  |  |  |  |  |  |  |  |  |  |  |  |  |  |  |  |  |  |  |
| TOTAL | 7.20 | 7.07 | 7.14 |  | 1.90 | 2.00 | 1.95 | 1.25 | 1.47 | 1.36 | 1.36 | 1.57 | 1.47 | 1.48 | 1.69 | 1.59 | 1.57 | 1.86 | 1.71 | 3.91 | 3.70 | 3.81 |  | 4.14 | 3.05 | 3.73 |  | 0.86 | 1.46 | 1.09 |
